# Gels: Synthesis, Characterization and Applications in High Performance Chemistry

**DOI:** 10.3390/gels9040287

**Published:** 2023-04-01

**Authors:** Viorel-Puiu Paun

**Affiliations:** 1 Department of Physics, Faculty of Applied Sciences, University Politehnica of Bucharest, 060042 Bucharest, Romania; viorel.paun@physics.pub.ro; 2 Academy of Romanian Scientists, 050094 Bucharest, Romania

Organogels, hydrogels, and ionic gels are investigated both theoretically and experimentally. Detailed research is focused on both their synthesis and applications in high-performance chemistry and its important branches. All of the abovementioned gels are characterized from structural and supramolecular points of view by FTIR, NMR, X-ray diffraction, and POM. The keywords proposed on this occasion are as follows: organogels, hydrogels, ionic gels, chitosan, fractal analysis; these terms are extremely pertinent to this research piece. As a result, all these reference themes, as well as those associated with them, are touched upon in this Editorial.

The articles in this selection are assumed to focus on one or more of the topics listed above. Now that we have in front of us all of the works published in the journal under the auspices of the Special Issue proposed by the Guest Editor, who later became the editor of this volume, we can consider the central topic the refined analysis of hydrogels, including, for example, the minting of coins on chitosan. The investigation of a hydrogel for wound-healing applications has been also well received. The biodegradation of the hydrogel has been monitored in media mimicking the wound exudate by gravimetrical measurements, SEM imaging, and fractal analysis of SEM and pictures. The images of interest could be evaluated by fractal analysis, calculating the fractal dimension and the lacunarity values as a quantitative measure of the homogeneity of the material and its texture through their topological analysis in some of the reference works. In addition, its biocompatibility, antimicrobial properties, and biodegradation have been considered in vitro in scientific papers that were referenced on this occasion and included in this volume.

The works edited in this book are the ones that appeared in the journal *Gels* in a Special Issue with the same name, more precisely the 16 distinct published papers plus an Editorial, signed by the editor of this volume.

An evaluation by multifractal analysis was applied to the images related to xerogel morphology details obtained by multifractal analysis and scanning electron microscopy. This work is the first introduced in this Editorial. The title of the work is “Xerogels Morphology Details by Multifractal Analysis and Scanning Electron Microscopy Images Evaluations of 5-Fluorouracil Release from Chitosan-Based Matrix”, the authors of which are Maria-Alexandra Paun, Mihai-Virgil Nichita, Vladimir-Alexandru Paun and Viorel-Puiu Paun. Four medicament delivery formulations based on 5-fluorouracil in a chitosan substantial matrix were realized in situ via 3,7-dimethyl-2,6-octadienal element hydrogelation. Representative samples of the final realized compounds were investigated from an analytic, constitutional, and morphological viewpoint via Fourier transform infrared (FTIR) spectroscopy and scanning electron microscopy (SEM). The SEM images of the formulations were investigated in concordance with fractal analysis, and the fractal dimensions and lacunarity were computed. The developed mathematical multifractal model is necessarily confirmed by the experimental measurements corresponding to the 5-fluorouracil release outside the chitosan-formed matrix [1].

The second work presented is named “A Modified Sol–Gel Synthesis of Anatase {001}-TiO_2_/Au Hybrid Nanocomposites for Enhanced Photodegradation of Organic Contaminants”, the authors of which are Abubakar Katsina Usman, Diana-Luciana Cursaru, Gheorghe Brănoiu, Raluca Şomoghi, Ana-Maria Manta, Dănuţa Matei and Sonia Mihai. A sol–gel synthesis technique was employed for the preparation of anatase phase {001}-TiO_2_/Au hybrid nanocomposites (NCs). A scalable, schematic, and cost-efficient method was successfully modified using HF and NH4OH capping agents. The photocatalytic activity of the as-synthesized {001}-TiO_2_/Au NCs was tested over two-cycle degradation of methylene blue (MB) dye and pharmaceutical active compounds (PhACs) of ibuprofen and naproxen under direct sunlight illumination at 35 °C and 44,000 lx. Transmission electron microscopy (TEM), high-resolution transmission electron microscopy (HR-TEM), fast Fourier transform (FFT), X-ray diffraction (XRD), X-ray photoelectron spectroscopy (XPS), energy dispersive X-ray spectroscopy (EDS), and ultraviolet–visible diffuse reflectance spectroscopy (UV–Vis DRS) were employed for the characterization of the as-prepared sample. The characterization results from the TEM, XPS, and XRD studies established both the distribution of Au colloids on the surface of TiO_2_ material and the presence of the highly crystalline structure of anatase {001}-TiO_2_/Au NCs. Photodegradation results from the visible light irradiation of MB indicate the enhanced photocatalytic performance of Au/TiO_2_ NCs over TiO_2_. The results from the photocatalytic activity test performed under direct sunlight exposure exhibited promising photodegradation efficiencies. In the first cycle, the sol–gel synthesized material exhibited relatively better efficiencies (91%) with the MB dye and ibuprofen, while the highest degradation efficiency for the second cycle was 79% for the MB dye. Pseudo first-order photodegradation rates from the first cycle were determined to be comparatively slower than those from the second degradation cycle [2].

The third work is entitled “Structural, Optical, and Sensing Properties of Nb-Doped ITO Thin Films Deposited by the Sol–Gel Method”, the authors of which are Madalina Nicolescu, Daiana Mitrea, Cristian Hornoiu, Silviu Preda, Hermine Stroescu, Mihai Anastasescu, Jose Maria Calderon-Moreno, Luminita Predoana, Valentin Serban Teodorescu, Valentin-Adrian Maraloiu, Maria Zaharescu and Mariuca Gartner. The aim of the present study was the development of Nb-doped ITO thin films for carbon monoxide (CO) sensing applications. The detection of CO is imperious because of its high toxicity, with long-term exposure having a negative impact on human health. Using a feasible sol–gel method, the doped ITO thin films were prepared at room temperature and deposited onto various substrates (Si, SiO_2_/glass, and glass). The structural, morphological, and optical characterization was performed by the following techniques: X-ray diffractometry (XRD), atomic force microscopy (AFM), scanning electron microscopy (SEM), transmission electron microscopy (TEM), and UV/Vis/NIR spectroscopic ellipsometry (SE). The analysis revealed a crystalline structure and a low surface roughness of the doped ITO-based thin films. XTEM analysis (cross-sectional transmission electron microscopy) showed that the film has crystallites of the order of 5–10 nm and relatively large pores (around 3–5 nm in diameter). A transmittance value of 80% in the visible region and an optical band-gap energy of around 3.7 eV were found for dip-coated ITO/Nb films on SiO_2_/glass and glass supports. The EDX measurements proved the presence of Nb in the ITO film in a molar ratio of 3.7%, close to the intended one (4%). Gas testing measurements were carried out on the ITO undoped and doped thin films deposited on glass substrate. The presence of Nb in the ITO matrix increases the electrical signal and the sensitivity to CO detection, leading to the highest response for a 2000 ppm CO concentration at a working temperature of 300 °C [3].

The fourth paper in this presentation is a study entitled “Low Release Study of Cefotaxime by Functionalized Mesoporous Silica Nanomaterials”, whose authors are Dan Eduard Mihaiescu, Daniela Istrati, Alina Moroșan, Maria Stanca, Bogdan Purcăreanu, Rodica Cristescu, Bogdan Ștefan Vasile and Roxana Doina Trușca. Here, a well-known antibiotic is discussed. As a third-generation β-lactam antibiotic, cefotaxime displays a broad spectrum with Gram-positive and Gram-negative bacteria activity and is included in the WHO’s essential drug list. In order to obtain new materials with sustained release properties, the present research focuses on the study of cefotaxime absorption and desorption from different functionalized mesoporous silica supports. The MCM-41-type nanostructured mesoporous silica support was synthesized by the sol–gel technique using a tetraethyl orthosilicate (TEOS) route and cetyltrimethylammonium bromide (CTAB) as a surfactant, at room temperature and normal pressure. The obtained mesoporous material (MCM-41 class) was characterized through nuclear magnetic resonance (NMR), scanning electron microscopy (SEM), high-resolution transmission electron microscopy (HR-TEM), N2 absorption–desorption (BET) and Fourier transform infrared spectroscopy (FT-IR), demonstrating a good micro-structured homogeneity (SEM images), a high surface area (BET, 1029 m^2^/g) correlated with high silanolic activity (Q3/Q4 peak ratio from 29Si MAS-NMR), and an expected uniform hexagonal structure (2–3 nm, HRTEM). In order to non-destructively link the antibiotic compound on the solid phase, MCM-41 was further functionalized in two steps: with aminopropyl trimethoxysilane (APTMS) and glutaraldehyde (GA). Three cefotaxime-loaded materials were comparatively studied for low release capacity: the reference material with adsorbed cefotaxime on MCM-41, MCM-41/APS (aminopropyl silyl surface functionalization) adsorbed cefotaxime material, and APTMS–GA bounded MCM-41—cefotaxime material. The slow-release profiles were obtained by using an on-flow modified HPLC system. A significant improved release capacity was identified in the case of MCM-41/APS/GA—cefotaxime due to the covalent surface grafting of the biological active compound, recommending this class of materials as an effective carrier of bioactive compounds in wound dressing, anti-biofilm coatings, advanced drugs, and other related applications [4].

The fifth work is called “TEGylated Phenothiazine-Imine-Chitosan Materials as a Promising Framework for Mercury Recovery”, and has the following authors: Sandu Cibotaru, Daniela Ailincai, Bianca-Iustina Andreica, Xinjian Cheng, and Luminita Marin. This paper reports new solid materials based on TEGylated phenothiazine and chitosan, with a high capacity to recover mercury ions from aqueous solutions. They were prepared by hydrogelation of chitosan with a formyl derivative of TEGylated phenothiazine, followed by lyophilization. Their structural and supramolecular characterization was carried out by 1H-NMR and FTIR spectroscopy, as well as X-ray diffraction and polarized light microscopy. Their morphology was investigated by scanning electron microscopy, and their photophysical behaviour was examined by UV/Vis and emission spectroscopy. Swelling evaluation in different aqueous media indicated the key role played by the supramolecular organization for their hydrolytic stability. Mercury recovery experiments and the analysis of the resulting materials by X-ray diffraction and FTIR spectroscopy showed a high ability of the studied materials to bind mercury ions by coordination with the sulfur atom of phenothiazine, imine linkage, and amine units of chitosan [5].

The sixth work in this suite is “Magnetic Application of Gadolinium Orthoferrite Nanoparticles Synthesized by Sol–Gel Auto-Combustion Method”, authored by Loganathan Guganathan, Chinnaiyan Rajeevgandhi, Kaliyamurthy Sathiyamurthy, Kokila Thirupathi, Madhappan Santhamoorthy, Ellappan Chinnasamy, Chaitany Jayprakash Raorane, Vinit Raj, Seong-Cheol Kim and Pichapillai Anand. In this manuscript, the synthesis of gadolinium orthoferrite nanoparticles using the sol–gel auto-combustion technique is presented. The obtained gadolinium orthoferrite nanoparticles were annealed at various temperatures, including 800 °C, 900 °C, 1000 °C, and 1100 °C. The synthesized materials were analyzed by various instrumental characterizations. The vibrational characteristics of the synthesized samples were verified by FTIR. The surface morphology of the gadolinium orthoferrite nanoparticles was analyzed by FE-SEM and HR-TEM, revealing their spherical structural morphology and uniform particle structure. The presence of the elemental features was analyzed in the gadolinium orthoferrite nanoparticles by EDAX. The surface analysis of the core ranges of the XPS-recorded spectra were obtained for the elemental states of the Gd, Fe, and O factors in the samples, and it additionally characterized the different levels of oxidative states by fitting the levels of the high-resolution parameters of Gd 4d, Fe 2p, and O 1s. The magnetic properties of the samples were investigated by VSM. The measurement of the magnetic parameters revealed that gadolinium orthoferrite nanoparticles exhibit a ferromagnetic nature [6].

The seventh paper is called “Photocatalytic and Antibacterial Properties of Doped TiO_2_ Nanopowders Synthesized by Sol−Gel Method”, authored by Silviu Preda, Jeanina Pandele-Cușu, Simona Viorica Petrescu, Elena Mădălina Ciobanu, Gabriela Petcu, Daniela C Culita, Nicoleta G. Apostol, Ruxandra M. Costescu, Iuliana Raut, Mariana Constantin and Luminița Predoană. For environmental applications, nanosized TiO_2_-based materials are known as the most important photocatalysts and are intensively studied for advantages including their higher activity, lower price, and chemical and photoresist properties. Zn- or Cu-doped TiO_2_ nanoparticles with anatase crystalline structures were synthesized by the sol−gel process. Titanium (IV) butoxide was used as a TiO_2_ precursor, with parental alcohol as a solvent, and a hydrolysing agent (ammonia-containing water) was added to obtain a solution with pH 10. The gels were characterized by TG/DTA analysis, SEM, and XPS. Based on TG/DTA results, a temperature of 500 °C was chosen for processing the powders in air. The structure of the samples thermally treated at 500 °C was analysed by XRD, and the patterns show crystallization in a single phase of TiO_2_ (anatase). The surface of the samples and the oxidation states were investigated by XPS, confirming the presence of Ti, O, Zn, and Cu. The antibacterial activity of the nanoparticle powder samples was verified using the Gram-positive bacterium Staphylococcus aureus. The photocatalytic efficiency of the doped TiO_2_ nanopowders for the degradation of methyl orange (MO) was examined here to evaluate the potential applications of these materials for environmental remediation [7].

The next work, eighth in order on the list, is “A Multifractal Vision of 5-Fluorouracil Release from Chitosan-Based Matrix”, authored by Maria-Alexandra Paun, Vladimir-Alexandru Paun, and Viorel-Puiu Paun. A suite of four drug-deliverance formulations grounded on 5-fluorouracil enclosed in a chitosan-founded intercellular substance was produced by 3,7-dimethyl-2,6-octadienal with in situ hydrogelation. The formulations were examined from a morphological and structural point of view by Fourier transform infrared (FTIR) spectroscopy and microscopy with polarized light. The polarized optical microscopy (POM) pictures of the three obtained representative formulations were investigated by fractal analysis. The fractal dimension and lacunarity of each of them were thus calculated. In this paper, a novel theoretical method for mathematically describing medicament deliverance dynamics in the context of the polymeric medicament constitution limit has been advanced. Assuming that the polymeric drug motion unfolds only on the so-called non-differentiable curves (considered mathematically multifractal curves), it appears that in a one-dimensional hydrodynamic movement within a multifractal formalism, the drug-release physics models are provided by isochronous kinetics, but at a scale of resolution necessarily non-differentiable [8].

The ninth paper presented here is “A Molecular Description of Hydrogel Forming Polymers for Cement-Based Printing Paste Applications”, with the following authors: Hajar Taheri-Afarani, Eugene Mamontov, William R. Carroll and Joseph J. Biernacki. This research endeavors to link the physical and chemical characteristics of select polymer hydrogels to differences in printability when used as printing aids in cement-based printing pastes. A variety of experimental probes including differential scanning calorimetry (DSC), NMR-diffusion ordered spectroscopy (DOSY), quasi-elastic neutron scattering (QENS) using neutron backscattering spectroscopy, and X-ray powder diffraction (XRD), along with molecular dynamic simulations, were used. Conjectures based on objective measures of printability and physical and chemical–molecular characteristics of the polymer gels are emerging that should help target printing aid selection and design and mix formulation. Molecular simulations were shown to link a higher hydrogen bond probability and larger radius of gyration to higher-viscosity gels. Furthermore, the higher-viscosity gels also produced higher elastic properties, as measured by neutron backscattering spectroscopy [9].

The tenth paper, entitled “Study on the Hydration Reaction of Typical Clay Minerals under Alkali and Sulfate Compound Activation” is authored by Siqi Zhang, Zeping Wu, Jiaming Chen, Runsheng Xu, Meina Wang, and Wen Ni. Sand, stone, tailings, and other aggregates often contain a small amount of clay mineral, and their hydration activity is low, thereby lowering the concrete performance indexes while negatively affecting their resource utilisation. In this study, clay minerals, calcium hydroxide, and desulfurised gypsum were used to prepare cementitious materials to examine kaolinite, montmorillonite, illite, and chlorite clay mineral contents under compound activation. The effects of the curing temperature and water reducer on clay samples were analysed. The results showed that the compressive strength of kaolinite samples cured at 25 °C and 55 °C reached 1.09 and 4.93 MPa in 28 days and increased by 43% and 12%, respectively, after adding a 0.3% water reducer. Montmorillonite was activated, and its compressive strength reached 5.33 MPa after curing at 55 °C in 28 days. Illite exhibited some activity and its compressive strength reached 1.43 MPa after curing at 55 °C in 28 days, and the strength increased slightly after adding a water reducer. The chlorite sample had no strength after activation under the same conditions. Furthermore, X-ray diffraction and scanning electron microscopy and energy-dispersive spectroscopy microstructure analyses showed that after alkali and sulfate activation, the hydration products of activated clay minerals mainly included ettringite, hydrated calcium aluminate, and hydrated calcium silicate. The increase in curing temperature accelerated the reaction speed and improved the early strength. However, the effect on chlorite minerals was not obvious [10].

The eleventh study, entitled “Synthesis and Characterization of Fly Ash-Based Geopolymers Activated with Spent Caustic”, was authored by Ruobing Zhang, Qian Wan, Yimin Zhang and Xuemian Zhang. The spent caustic with strong alkali first replaced the alkali activator to prepare the geopolymer. The influence of spent caustic to the geopolymer was characterized through compressive strength measurement, XRD, MIP analysis and NMR, and the immobilization efficiency of organics in geopolymer was evaluated through the measurement of the total organic carbon (TOC). The results show that the spent caustic can partially replace the alkali activator to prepare the geopolymer, and it shows a better performance than that activated with pure NaOH solution when the alkalinity was between 4 mol and 14 mol. The organic matter in the spent alkali can be effectively fixed in the geopolymer, which will hinder the geopolymerization in the initial stage of the polymerization reaction but has little effect on the chemical structure and mechanical properties of the final product. With the degree of alkalinity increasing, the immobilization efficiency is improved, and the maximum can reach 84.5%. The organics in the spent caustic will hinder geopolymerization at the initial stage but have little effect on the chemical structure and mechanical property of the final product. This study proposes a new method for the recycling of spent caustic, which also reduces the preparation cost of geopolymers [11].

The twelfth paper is entitled “Hydrogel Beads of Amidoximated Starch and Chitosan as Efficient Sorbents for Inorganic and Organic Compounds”, with the authors listed as follows: Diana Felicia Loghin, Melinda Maria Bazarghideanu, Silvia Vasiliu, Stefania Racovita, Marius-Mihai Zaharia, Tudor Vasiliu, and Marcela Mihai. The synthesis of hydrogel beads involving natural polymers is, nowadays, a leading research area. Among natural polymers, starch and chitosan represent two biomolecules with proof of efficiency and a low economic impact in various utilization fields. Therefore, herein, the features of hydrogel beads obtained from chitosan and three sorts of starch (potato, wheat, and rice starches), grafted with acrylonitrile and then amidoximated, were extensively investigated for their use as sorbents for heavy-metal ions and dyes. The hydrogel beads were prepared by ionotropic gelation/covalent cross-linking of chitosan and functionalized starches. The chemical structure of the hydrogel beads was analyzed by FT-IR spectroscopy; their morphology was revealed by optical and scanning electron microscopies, while the influence of the starch functionalization strategies on the crystallinity changes was evaluated by X-ray diffraction. Molecular dynamics simulations were used to reveal the influence of the grafting reactions and grafted structure on the starch conformation in solution and their interactions with chitosan. The sorption capacity of the hydrogel beads was tested in batch experiments, as a function of the beads’ features (synthesis protocol, starch sort) and simulated polluted water, which included heavy metal ions (Cu^2+^, Co^2+^, Ni^2+^ and Zn^2+^) and small organic molecules (Direct Blue 15 and Congo red) [12].

We now present paper number thirteen, “Photopolymerizable Ionogel with Healable Properties Based on Dioxaborolane Vitrimer Chemistry”, whose authors are Fengdi Li, Giao T. M. Nguyen, Cédric Vancaeyzeele, Frédéric Vidal and Cédric Plesse. Ionogels are solid polymer gel networks loaded with ionic liquid (IL) percolating throughout each other, giving rise to ionically conducting solid electrolytes. They combine the mechanical properties of polymer networks with the ionic conductivity, non-volatility, and non-flammability of ILs. In the frame of their applications in electrochemical-based flexible electronics, ionogels are usually subjected to repeated deformation, making them susceptible to damage. It appears critical to devise a simple and effective strategy to improve their durability and lifespan by imparting them with healing ability through vitrimer chemistry. In this work, we report the original in situ synthesis of polythioether (PTE)-based vitrimer ionogels using fast photopolymerization through thiol-acrylate Michael addition. PTE-based vitrimer was prepared with a constant amount of the trithiol crosslinker and varied proportions of static dithiol spacers and dynamic chain extender BDB containing dynamic exchangeable boronic ester groups. The dynamic ionogels were prepared using 50 wt% of either 1-Ethyl-3-methylimidazolium bis(trifluoromethylsulfonyl) imide or 1-Ethyl-3-methylimidazolium trifluoromethanesulfonate, both of which were selected for their high ionic conductivity. They are completely amorphous (*T_g_* below −30 °C), suggesting they can be used at low temperatures. They are stretchable with an elongation at break around 60%, soft with a Young’s modulus between 0.4 and 0.6 MPa, and they have high ionic conductivities for solid-state electrolytes in the order of 10^−4^ S·cm^−1^ at room temperature. They display dynamic properties typical of the vitrimer network, such as stress relaxation and healing, retained despite the large quantity of IL. The design concept illustrated in this work further enlarges the library of vitrimer ionogels and could potentially open a new path for the development of more sustainable, flexible electrochemical-based electronics with extended service life through repair or reprocessing [13].

The fourteenth paper is called “Hybrid Epoxy-Alkyl Sol–Gel Coatings Reinforced with SiO_2_ Nanoparticles for Corrosion Protection of Anodized AZ31B Mg Alloy”; its authors are Emilia Merino, Alicia Durán, Silvia Ceré, and Yolanda Castro. AZ31B Mg alloys were anodized at different potentials using an alkaline electrolyte. Then, an epoxy-alkyl silane sol reinforced with SiO_2_ nanoparticles was prepared by sol–gel and deposited on top of the optimized anodic layers. 1-Methyl imidazole was added to the sol to promote a partial epoxy ring aperture and improve the condensation degree of the inorganic network. The results showed that the curing temperature affects the inorganic polycondensation of the organic–inorganic network; this effect was analyzed by ^29^Si and ^13^C solid-state NMR spectroscopy. Electrochemical impedance spectroscopy in 3.5 wt% NaCl solution revealed that the corrosion resistance is enhanced by the anodized process obtained for Mg alloy anodized at 100 V/2 min. However, quick deterioration of the oxide film with immersion time was evident, showing a reduction in the protection efficiency (ηE%) of 76.5% after 16 h/immersion. The deposition of an epoxy-alkyl coating improved the ηE% by up to 98.6% after 72 h/immersion. The proposed hybrid coating used for post-sealing the porous anodized Mg alloy looks to be a good alternative protective barrier to control the corrosion process of Mg alloys. A suitable compromise between the cross-linking network and curing temperature is necessary to obtain a good barrier coating [14].

The penultimate paper presented, the fifteenth, is entitled “Biocompatible Chitosan-Based Hydrogels for Bioabsorbable Wound Dressings”, and has the following authors: Ramona Lungu, Maria-Alexandra Paun, Dragos Peptanariu, Daniela Ailincai, Luminita Marin, Mihai-Virgil Nichita, Vladimir-Alexandru Paun, and Viorel-Puiu Paun. Supramolecular hydrogels based on chitosan and monoaldehydes are biomaterials with high potential for a multitude of bioapplications. This is due to the proper choice of the monoaldehyde that can tune the hydrogel properties for specific practices. In this conceptual framework, the present paper deals with the investigation of a hydrogel used as a bioabsorbable wound dressing. To this aim, chitosan was cross-linked with 2-formylphenylboronic acid to yield a hydrogel with antimicrobial activity. FTIR, NMR, and POM procedures have characterized the hydrogel from a structural and supramolecular point of view. At the same time, its biocompatibility and antimicrobial properties were also determined in vitro. Furthermore, in order to assess the bioabsorbable character, its biodegradation was investigated in vitro in the presence of lysosome in media of different pH values, mimicking the wound exudate at different stages of healing. The biodegradation was monitored by gravimetrical measurements, SEM microscopy, and fractal analyses of the images. The fractal dimension values and the lacunarity of SEM pictures were accurately calculated. All these successful investigations led to the conclusion that the tested materials are at the expected high standards [15].

The sixteenth paper and final paper, entitled “Analysis of Three-Dimensional Cell Migration in Dopamine-Modified Poly(aspartic acid)-Based Hydrogels”, is authored by David Juriga, Eszter Eva Kalman, Krisztina Toth, Dora Barczikai, David Szöllősi, et al. Several types of promising cell-based therapies for tissue regeneration have been in development worldwide. However, for the successful therapeutical application of cells in this field, appropriate scaffolds are also required. Recently, the research for suitable scaffolds has focused on polymer hydrogels due to their similarity to the extracellular matrix. The main limitation regarding amino-acid-based hydrogels is their difficult and expensive preparation, which can be avoided by using poly(aspartamide) (PASP)-based hydrogels. PASP-based materials can be chemically modified with various bioactive molecules for the final application purpose. In this study, dopamine containing PASP-based scaffolds is investigated, since dopamine influences several cell biological processes, such as adhesion, migration, proliferation, and differentiation, according to the literature. Periodontal ligament cells (PDLCs) of neuroectodermal origin and the SH-SY5Y neuroblastoma cell line were used for in vitro experiments. The chemical structure of the polymers and hydrogels was proved by ^1^H-NMR and FTIR spectroscopy. Scanning electron microscopy (SEM) images confirmed the suitable pore size range of the hydrogels for cell migration. Cell viability assay was carried out according to a standardized protocol using the WST-1 reagent. To visualize the three-dimensional cell distribution in the hydrogel matrix, two-photon microscopy was used. According to our results, dopamine containing PASP gels can facilitate vertical cell penetration from the top of the hydrogel at a depth of around four cell layers (~150 μm). To quantify these observations, a detailed image analysis process was developed and first introduced in this paper [16].

To conclude this article, we would like to express our appreciation for the papers contained in this book, sixteen in number, focused on extraordinary applications of gels for pharmaceutical uses, drug release, oncologic cure, new food production, and careful monitoring of radiation, having, as stated, the intent to ameliorate the quality of life in people of modern society.
